# Hydrogel Nanocomposite-Derived Nickel Nanoparticles/Porous Carbon Frameworks as Non-Precious and Effective Electrocatalysts for Methanol Oxidation

**DOI:** 10.3390/gels8090542

**Published:** 2022-08-29

**Authors:** Hamud A. Altaleb, Abdulwahab Salah, Badr M. Thamer

**Affiliations:** 1 Department of Chemistry, Faculty of Science, Islamic University of Madinah, Madinah 42351, Saudi Arabia; 2 Faculty of Chemistry, Northeast Normal University, Changchun 130024, China; 3 Chemistry Department, Science College, King Saud University, Riyadh 11451, Saudi Arabia

**Keywords:** hydrogel, nickel nanoparticles, porous carbon frameworks, methanol oxidation, electrocatalyst

## Abstract

Innovative and facile methods for the preparation of metal nanoparticles (MNPs) with A highly uniform distribution and anchored on a unique substrate are receiving increasing interest for the development of efficient and low-cost catalysts in the field of alternative and sustainable energy technologies. In this study, we report a novel and facile metal-ions adsorption-pyrolysis method based on a hydrogel nanocomposite for the preparation of well-distributed nickel nanoparticles on 3D porous carbon frameworks (Ni@PCFs). The pyrolysis temperature effect on electrocatalytic activity toward methanol oxidation and catalyst stability was investigated. Physicochemical characterizations (SEM, TEM, and XRD) were used to determine the morphology and composition of the prepared electrocatalyst, which were then linked to their electrocatalytic activity. The experimental results indicate that the catalyst synthesized by pyrolysis at 800 °C (Ni@PCFs-8) exhibits the highest electrocatalytic activity for oxidation of methanol in alkaline media. Additionally, prepared Ni@PCFs-8 displays a remarkable increase in electrocatalytic activity after activation in 1 M KOH and excellent stability. The adsorption-pyrolysis pathway ensures that the Ni NPs are trapped in the PCFs, which can provide highly reactive surface sites. This work may provide a facile and effective strategy for preparing uniformly distributed metallic NPs on a 3D PCF substrate with high catalytic activity for energy applications.

## 1. Introduction

Rising global energy demand and climate change are among the biggest future challenges facing the countries of the world [1,2]. Researchers have been making great efforts to find alternative and sustainable energy sources with a cost competitive to energy derived from fossil fuels [3]. For example, fuel cells are one of these alternatives and one of the sustainable energy sources that will play a significant role in providing energy for a variety of applications [4]. Fuel cells are distinguished by the variety of fuels that can be used in them, including hydrogen, methanol, ethanol, urea, formaldehyde, glycol, glycerol, formic acid, and others [5]. Methanol-based fuel cells are one of the most common types because methanol is easy to store, has low emissions, can be obtained from biomass, and has an energy density of 6.09 kW h kg^−1^. Direct methanol fuel cells (DMFs) consist of three major components: a cathode electrode, anode electrode, and separation membrane. The anode electrode is an important component in determining the efficiency and cost of DMFs [6,7]. Materials based on noble metals as the anode electrodes are typically utilized, despite their scarcity and high cost. Massive efforts have been made to replace noble metal-based electrocatalysts with low-cost and more efficient materials such as non-precious transition metals [8,9]. Among the transition metals used for this purpose, Ni [10,11], Mn [12], Mo [13], Cu [14], and Co [15,16] are the most common. Nickel-based materials have received increasing attention due to their high catalytic efficiency and high stability in alkaline media [17,18,19,20,21]. Many studies have demonstrated that the morphology and size of metal particles and their supporting material play a pivotal role in their electrocatalytic performance [22,23,24]. Therefore, metal nanoparticles (MNPs) have received a lot of attention in recent decades due to their unique properties and structure. Highly active sites and a large surface area on the surface of MNPs contribute significantly to accelerating reactions and increasing product yield. Several methods for the synthesis of MNPs have been reported, including impregnation [25], supercritical fluid [26], flam spray pyrolysis [27], hydrothermal [28] and electrodeposition [29]. Some of these methods, such as impregnation, are simple and easy to use, but it is difficult to control the size of the MNPs and obtain small sizes with a narrow distribution. The integration of the impregnation and pyrolysis method is a promising route for producing MNPs with a supported carbon structure [30]. A few studies have succeeded in preparing MNPs dispersed in a carbon matrix by impregnation of metal salts into the biomass matrix, followed by pyrolysis [31,32,33]. In spite of these advancements, impregnation-pyrolysis requires optimization of the impregnated metallic salt ratio to avoid MNP agglomeration during the pyrolysis process and is a time-consuming process [34]. These drawbacks limit the strategy’s widespread application. As a result, a simple and convenient method for synthesizing small MNPs supported on carbon with a uniform distribution must be developed. Adsorption-pyrolysis is a promising method for overcoming agglomeration because the adsorbent adsorbs a specific mass of metal ions with a uniform distribution across its surface. Until now, no study has reported the synthesis of Ni NPs supported on porous carbon frameworks (PCFs) by the adsorption-pyrolysis method with a uniform distribution and exceptional electrocatalytic activity for methanol oxidation in alkaline media.

Herein, this study explores a facile and convenient strategy for synthesizing uniformly distributed nickel NPs anchored on 3D porous carbon frameworks (Ni@PCFs) through the pre-adsorption of Ni ions onto the surface of hydrogel nanocomposites, followed by pyrolysis at different temperatures. The prepared Ni@PCFs catalysts exhibit excellent electrocatalytic properties for methanol oxidation in alkaline media. This strategy is promising for the preparation of other metallic NPs with uniform distribution and high electrocatalytic performance due to its simple synthesis process and morphology of the catalyst support and the size of the MNPs are easy to control.

## 2. Results and Discussion

### 2.1. Characterization

The pure hydrogel was prepared by an in-situ grafting process and was made up of chitosan grafted to polyacrylic acid and its composite was prepared by incorporating electrospun carbon nanofibers (ECNFs) into the hydrogel matrix. The prepared hydrogel nanocomposite was used for nickel ion adsorption from an aqueous solution, followed by pyrolysis at different temperatures to produce the electrocatalyst, which was composed of nickel nanoparticles supported on porous carbon frameworks (Ni@PCFs).

**Figure 1:**
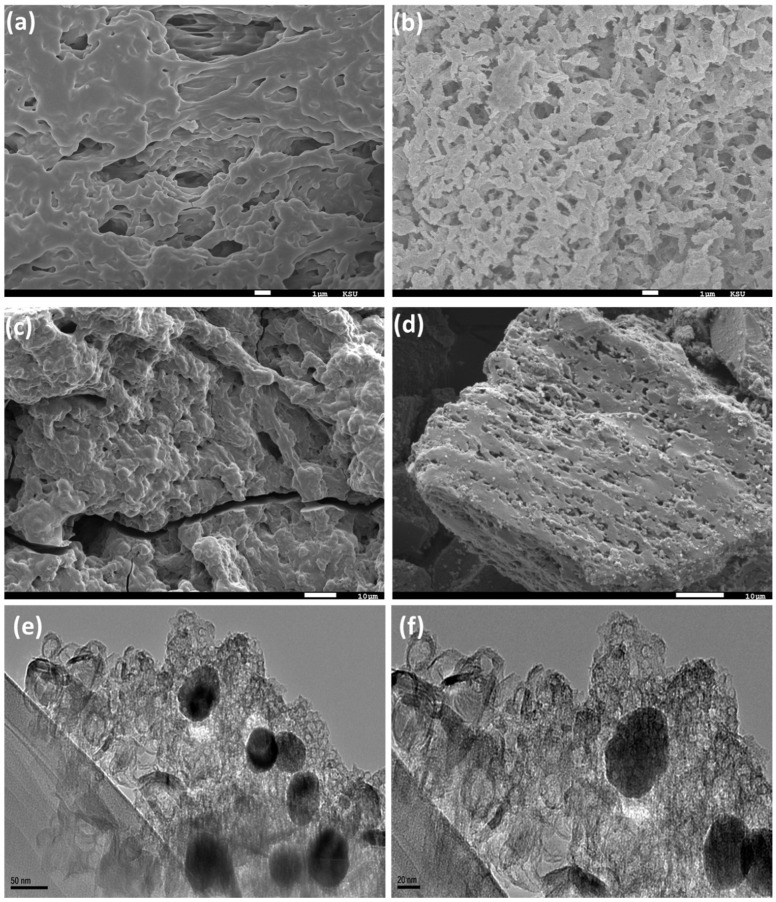
SEM images of (**a**) pure hydrogel, (**b**) hydrogel nanocomposite, (**c**) hydrogel nanocomposite/Ni^2+^ and (**d**) Ni@PCFs-8 catalyst and (**e**,**f**) TEM images of Ni@PCFs-8 catalyst.

The morphology of the hydrogel composite before and after the adsorption of nickel ions was investigated by SEM, as displayed in Figure 1a–c. The morphology of the pure hydrogel appeared in the form of a 3D structure with a smooth surface due to the crosslinking between the chitosan and polyacrylic acid chains. After the incorporation of ECNFs, the morphology became more porous, with a rough surface. The morphology of the ECNFs is shown in Figure S1. The adsorption of nickel ions by the hydrogel nanocomposite from the aqueous solution led to a change in its morphology and it became more wrinkled due to the swelling, indicating that the pores were filled with nickel ions. Figure 1d and Figure S2 show that the morphology of the Ni@PCFs had a three-dimensional porous carbon structure, with a distribution of Ni NPs on the surface and between the stacked layers that were supported by ECNFs. The TEM images (Figure 1e,f) display that the Ni NPs were distributed over the surface of the PCFs and fixed to it by a particle size fraction incorporated into the carbon structure.

**Figure 2:**
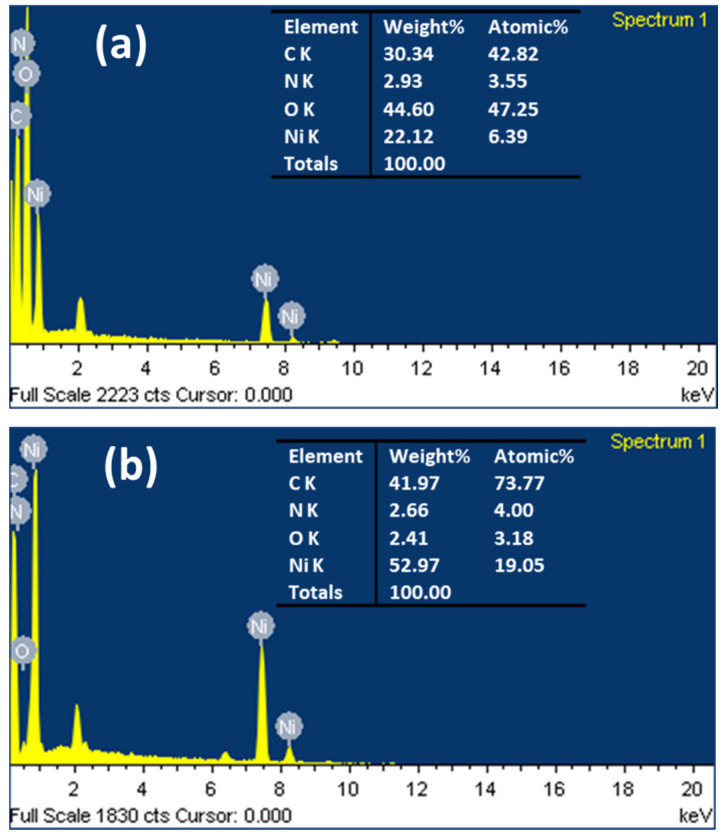
EDX analysis of the (**a**) hydrogel nanocomposite/Ni^2+^ and (**b**) Ni@PCFs-8.

**Figure 3:**
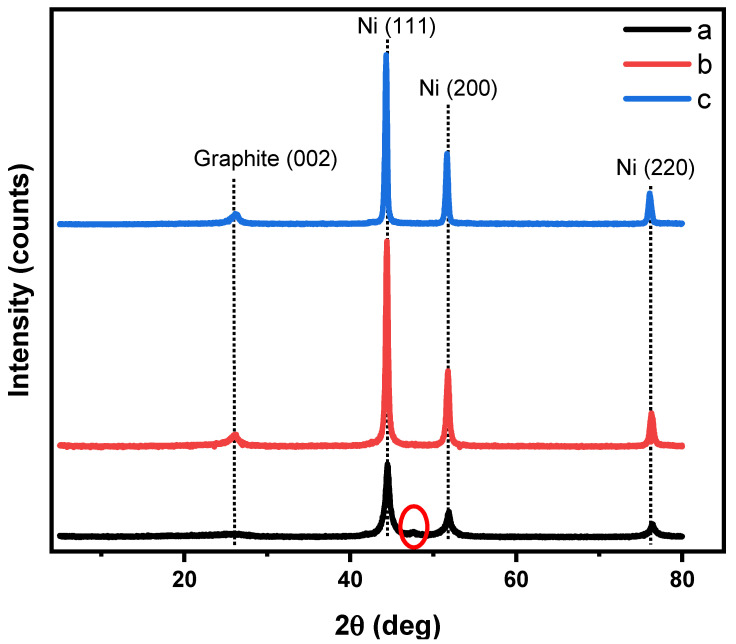
XRD patterns of (a) Ni@PCFs-6, (b) Ni@PCFs-7, and (c) Ni@PCF-8.

Energy dispersive X-ray analysis (EDX) confirmed the adsorption of nickel ions by the hydrogel nanocomposite, where the nickel weight ratio was 22% and increased to 53% after calcination due to the loss of oxygen, carbon, and nitrogen elements in the form of gases, as displayed in Figure 2. The EDX area and corresponding data of the hydrogel nanocomposite/Ni^2+^ and Ni@PCFs-8 catalyst are displayed in Figures S3 and S4. Figure 3 is showed X-ray diffraction patterns (XRD) of Ni@PCFs-6, Ni@PCFs-7, and Ni@PCFs-8. The XRD pattern of Ni@PCFs-6 exhibited three characteristic peaks at 76.42°,51.83° and 44.50°, which are assigned to the (220) (200) and (111) reflection of fcc Ni NPs [11]. Similarly, the same peaks appeared for the Ni@PCFs-7 and Ni@PCFs-8 samples, with a slight shift to lower values of 2θ with increasing calcination temperatures. It is worth noticing that the Ni@PCFs-6 sample had a peak at 47.5°, which is attributable to nickel’s hexagonal closed-packed (hcp) structure, but this peak was absent from the other samples. In contrast, the broad peak of graphite at 26° was weak in the Ni@PCFs-6 sample, but it was clear and detectable in the other samples. This result confirms that the increase in the temperature of calcination from 600 °C to 800 °C contributed to the formation of an ordered graphitic structure. As shown in Table 1, the graphitization percentage increased from 0 for the Ni@PCFs-6 sample to 36.86% for the Ni@PCFs-8 sample. This finding confirmed that the high pyrolysis temperature was critical in determining the nature of the carbon as a supported catalyst for the Ni NPs. It is well known that a higher degree of graphitization can improve the conductivity of carbon materials [35]. As a result, the pyrolysis of hydrogel-Ni ion at 800 °C is a powerful route to enhance the electrical characteristics of carbon as a support for the catalyst as well as improving interactions with Ni NPs. It is noteworthy that the nickel oxide was not detected by XRD, which indicates that the nickel ions had been completely reduced during the pyrolysis. From the Ni@PCFs-6 to the Ni@PCFs-7 and Ni@PCFs-8 samples, the half maximum (FWHM) of the peak at 44.50° became sharper, indicating that the crystallite sizes of the samples were different. The average sizes of the crystallites were calculated by Scherrer equation and to be 9.5, 15.21, and 22.84 nm from the main diffraction peaks of the fcc nickel plan for the Ni@PCFs-6, Ni@PCF-7, and Ni@PCF-8, respectively. Based on the EDX and XRD results, the prepared catalyst composition was composed of Ni NPs incorporated onto nitrogen-doped PCFs, with some oxygenated functional groups. The doped nitrogen improved the conductivity of the carbon support and charge transfer, while the oxygenated functional groups improved interactions at the nickel-support interface, surface wettability, and methanol molecule diffusion on the catalyst surface [36,37].

**Figure 4:**
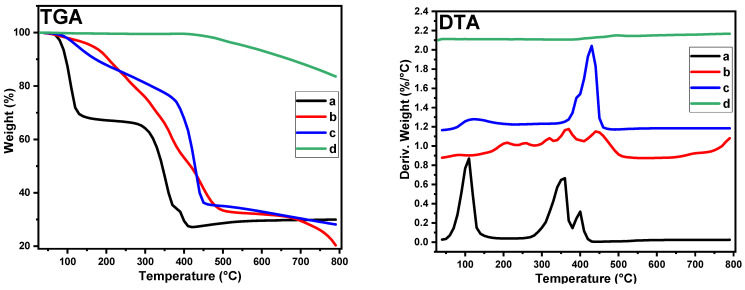
TGA/DTA analysis of (a) NiAc, (b) pure hydrogel, (c) Ni/hydrogel composite and (d) Ni@PCFs-8 catalyst.

Studying the pyrolysis of the metal nanoparticle precursor and substrate precursor by TGA is a proactive method for selecting the appropriate pyrolysis conditions for catalyst preparation. Figure 4 displays the thermal decomposition of nickel acetate tetrahydrate, pure hydrogel, and hydrogel composite/Ni^2+^. As shown in the weight loss ratio and weight loss derivative curves, the thermal decomposition of nickel acetate under nitrogen was carried out in three main steps: The first one took place in the range of 50–140 °C and peaked at 110 °C and is assigned to the dehydration of crystallized water (weight loss was 31%). The second step, which appeared between 270–355 °C and peaked at 355 °C, is assigned to the dehydrated intermediate decomposition (weight loss was 32%). The peak at 400 °C represents the third step and indicates the process of reducing nickel ions to Ni NPs. At 430 °C, the residual weight percentage was 27%, which is greater than the theoretical weight percentage of nickel (23.6%) in nickel acetate tetrahydrate. The increase in the residual weight was due to the presence of carbon residues with nickel particles. Thermal analysis of the nickel acetate as a source of nickel indicated that the appropriate pyrolysis temperature for nickel reduction during thermal decomposition under nitrogen gas was higher than 430 °C. Thermal decomposition of the hydrogel took place in multiple steps due to the multiplicity of its functional groups and the presence of crosslinking between its components. On the other hand, the thermal decomposition of the Ni/hydrogel composite occurred in two main steps: The first step took place in the range of 70 to 170 °C, and the percentage of weight lost was 10%. The main step occurred in the range 343–479 °C and peaked at 430 °C, with an estimated weight loss of 55%. The difference in thermal behavior between the hydrogel and Ni/hydrogel composite confirmed the adsorption of nickel ions by the hydrogel. Aside from that, the functional groups of the hydrogel interacted with the nickel ions. In contrast, the prepared catalyst (Ni@PCFs-8) showed high thermal stability in the range 30–500 °C, where the weight loss ratio did not exceed 3% at 500 °C.

### 2.2. Electrocatalytic Activity of the Ni@PCFs Catalyst

**Figure 5:**
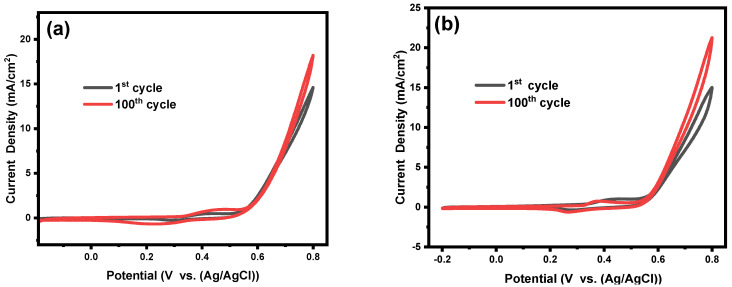

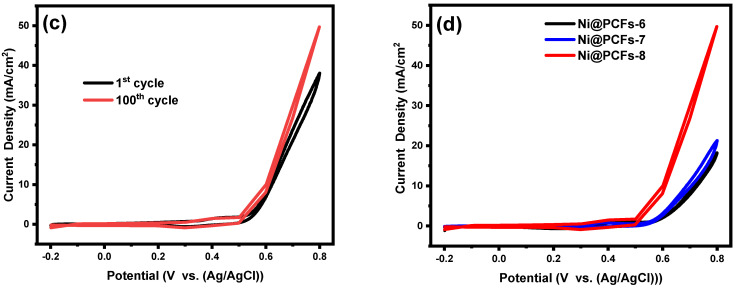
1st cycle and 100th CV of the (**a**) Ni@PCFs-6, (**b**) Ni NPs@C-7, (**c**) Ni@PCFs-8, and (**d**) cyclic voltammograms for all samples after 100th cycle, recorded at 0.05 V/s in 1.0 M of KOH solution.

After the physicochemical characterization, cyclic voltammetry (CV) was used to investigate the electrocatalytic activity of the prepared electrocatalysts towards oxidation of methanol in 1 M of KOH. Initially, 100th CV cycles were performed to activate and stabilize the electrocatalysts. The surface activation for Ni@PCFs-6, Ni@PCFs-7, and Ni@PCFs-8 catalyst was performed in alkaline media (1M of KOH) with a scan rate of 0.5 V/s. In the alkaline medium, a thin layer of NiOOH formed on the surface of the nickel NPs due to the oxidation process by OH^-^ anions. Figure 5 shows that all the samples had a peak at 0.45 V on the anodic scan and a peak at 0.26 V on the cathodic scan, showing the oxidation/reduction of Ni(OH)_2_/NiOOH. Moreover, during the anodic scan, a second sharp increase in the current density at 0.5 V vs. Ag/AgCl was attributed to the oxygen evaluation reaction (OER). The first CV cycle of Ni@PCFs-8 (37.91 mA/cm^2^) displayed a higher OER current density than Ni@PCFs-6 (14.53 mA/cm^2^) and Ni@PCFs-7 (14.90 mA/cm^2^). After 100 cycles, it was observed that the electrocatalytic activity was higher and that the OER current density was 18.15, 21.13, and 49.57 mA/cm^2^ for Ni@PCFs-6, Ni@PCFs-7, and Ni@PCFs-8, respectively. This result indicates that surface activation of the prepared catalysts occurred.

**Figure 6:**
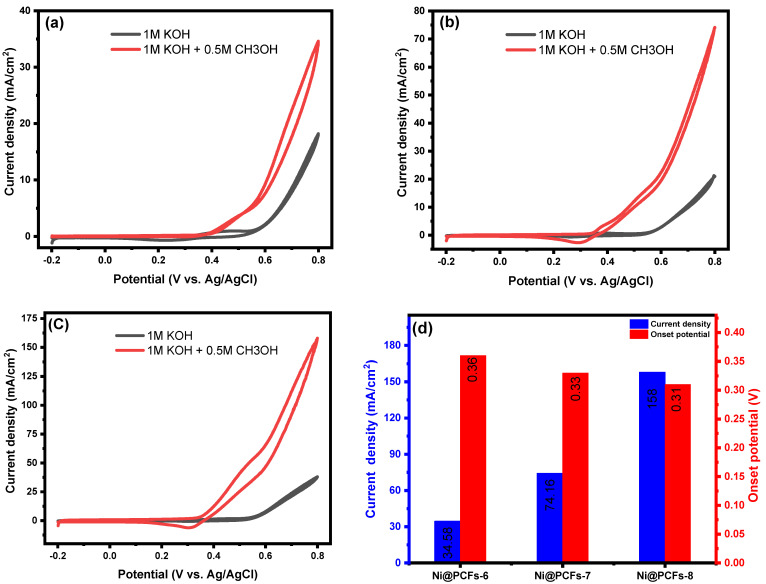
Cyclic voltammograms of the (**a**) Ni@PCFs-6, (**b**) Ni@PCFs-7, and (**c**) Ni@PCFs-8 recorded at 50 mV/s in the absence and presence of 0.5 M methanol in 1.0 M KOH solution and (**d**) steady state methanol oxidation current density and onset potential for the different studied electrocatalysts.

The onset potentials and anodic current densities are the two most important parameters that determine an electrocatalyst’s electrocatalytic activity. In the case of methanol oxidation, an effective electrocatalyst must have a low-onset potential and high current density. Figure 6a–c displays the recorded CV curves for Ni@PCFs-6, Ni@PCFs-7, and Ni@PCFs-8 with 0.5 M CH_3_OH in 1 M KOH electrolyte solution. At 0.5 M methanol concentration, the prepared electrocatalysts showed high electrocatalytic activity towards oxidation of methanol. The carbonization temperature used in the preparation of the electrocatalysts had a major effect on the electrocatalytic performance. As shown in Figure 6a–c, the anodic current densities of Ni@PCFs-6, Ni@PCFs-7, and Ni@PCFs-8 at 0.8 V were 34.58, 74.16, and 158 mA/cm^2^, respectively. Although the Ni NP sizes increased with increasing carbonization temperatures, the electrocatalytic activity increased. This result indicates that there was another critical factor other than the size of the Ni NPs—the conductivity of carbon substrate, which increased with increases in the temperature of carbonization process. On the other hand, it was noticed that the onset potential also decreased with increasing carbonation temperatures, where the onset potential values were 0.36, 0.33, and 0.31 for Ni@PCFs-6, Ni@PCFs-7, and Ni@PCFs-8, respectively—as displayed in Figure 6d. Additionally, in comparison with some nickel nanoparticle-based electrocatalysts, it seems clear that the prepared electrocatalyst can act as a comparable in methanol electrooxidation, as shown in Table 2. The effect of methanol concentration (0.1 to 2 M methanol solution) on the electrocatalytic performance of the prepared catalysts was also studied, as displayed in Figure S5. The current density increased as the methanol concentration electrolyte was increased, until it reached 0.5 M, which indicated the best electrocatalytic performance of all the samples. The current density declined to a lower value when the methanol concentration was higher than 0.5 M, indicating the existence of partially oxidized organic residue. Moreover, the increased methanol concentration may cause a higher surface coverage of Ni NPs with methanol and its intermediates, causing a blocking of OH^−^ from reaching the reactive sites on the catalyst [38].

**Figure 7:**
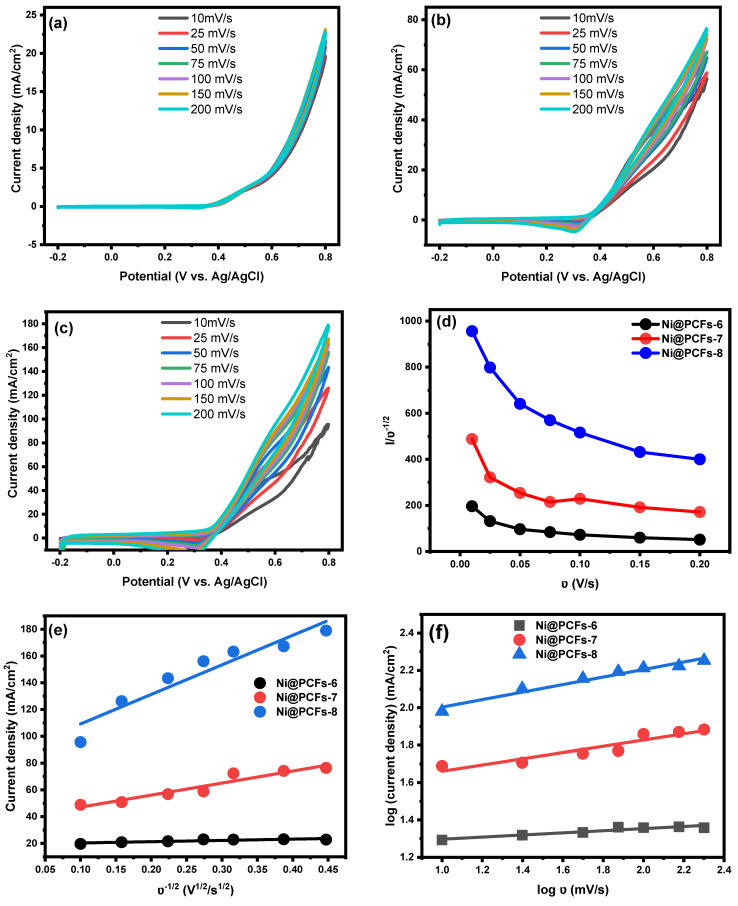
Effect of the scan rate on the electrooxidation of methanol in 1.0 M of KOH at the (**a**) Ni@PCFs-6, (**b**) Ni@PCFs-7, (**c**) Ni@PCFs-8. (**d**) The plot of methanol oxidation peak current density/ν^1/2^ values as a function of the ν. (**e**) The plot of methanol oxidation peak current density values as a function of the ν^1/2^. (**f**) The plot of the logarithm of methanol oxidation peak current density values as a function of the logarithm of ν^1/2^.

The catalytic kinetics of Ni@PCFs-6, Ni@PCFs-7, and Ni@PCFs-8 for the methanol electrooxidation reaction were studied at various scan rates from 10 to 200 mV/s in the presence of 0.5 M methanol in 1 M KOH solution. With increasing scan rates, the methanol oxidation peaks showed higher current densities in all the studied Ni@PCFs electrocatalysts. The current density increase varied by the type of catalyst; for example, when the scan rate was raised from 10 to 200 mV/s, the current density increased from 56.40 to 76.44 mA/cm^2^ and 95.58 to 178.97 mA/cm^2^ for the Ni@PCFs-7 and Ni@PCFs-8 catalyst, respectively. In contrast, the magnitude of the increase in current density was slight for Ni@PCFs-6 (19.62 to 22.83 mA/cm^2^) when the scan rate was raised from 10 to 200 mV/s. Figure 7e displays a plot of peak current density against the square root of the scan rate (ν^1/2^). Based on the straight linear relationship between ν^1/2^ and anodic current density, the oxidation of methanol on the surface of all the prepared catalysts was predicted to be carried out by the diffusion-controlled process. Similarly, the linear relationship between the logarithm of the current density and the logarithm of the scan rate demonstrates that the diffusion mechanism controlled the oxidation of methanol on the surface of the produced catalysts.

**Figure 8:**
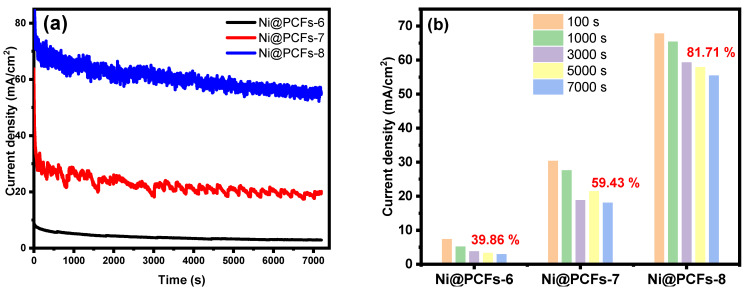
(**a**) Chronoamperograms of the Ni@PCFs-6, Ni@PCFs-7, and Ni@PCFs-8 electrocatalysts at a potential value of 0.7 V in (0.5 M methanol + 1.0 M KOH) solution for 7200 s. (**b**) Percentage of current density at different times.

The long-term stability of electrocatalysts is critical for practical applications. The long-term stability of the Ni@PCFs-6, Ni@PCFs-7, and Ni@PCFs-8 catalysts was examined using chronoamperometry at 0.7 V vs; Ag/AgCl electrode in 1.0 M KOH containing 0.5 M methanol for 7200 s.For the first few seconds, almost all of the catalysts exhibited a quick decline in current density, which was most likely due to the double-layer discharge and adsorbed poisoned intermediate species on the active sites. Following that, the current density eventually became pseudo-steady. The pseudo-steady current density of the Ni@PCFs-8 catalyst was substantially higher than that of the other catalysts. After 7200 s, the remaining current density of the Ni@PCFs-8 catalyst (54.97 mA/cm^2^) was 2.7 and 19.1-times than that of the Ni@PCFs-7 (20.13 mA/cm^2^) and Ni@PCFs-6 (2.88 mA/cm^2^) catalysts, respectively. Figure 8b exposes the pseudo-steady current density percentage of the catalysts at 100, 1000, 3000, 5000, and 7000 s. At 7000 s, the pseudo-steady current density percentage of the Ni@PCFs-6, Ni@PCFs-7, and Ni@PCFs-8 catalysts were 39.86, 59.43, and 81.71%, respectively. These findings suggest that the Ni@PCFs-6 catalyst was more durable with respect to the methanol oxidation reaction than other catalysts. The contact between the Ni NPs and the 3D PCF support, which was at its greatest when the carbonization temperature was 800 °C, was responsible for this stability.

## 3. Conclusions

In conclusion, we have developed a novel, low-cost, environmentally friendly, and particularly facile adsorption-pyrolysis method for the fabrication of Ni NPs supported on 3D porous carbon frameworks as non-precious electrocatalysts with exceptional electrocatalytic activity and durability for methanol oxidation. Nickel ion adsorption and dispersion across the whole surface of the hydrogel composite is a successful approach because it prevents Ni NPs aggregation during pyrolysis and does not require pre-optimization for the amount of metal precursor added. The resulting hydrogel-derived PCFs exhibit homogeneous microstructure with Ni NPs. The pyrolysis temperature plays a pivotal role in the electrocatalytic activity. The Ni@PCFs-8 catalyst showed superior electrocatalytic activity and outstanding stability for the methanol oxidation in alkaline media. Additionally, the Ni@PCFs-8 exhibited a more negative onset potential (0.31 V), with a high current density (158 mA/cm^2^) towards methanol oxidation at 0.8 V vs. Ag/AgCl. Furthermore, after 7000 s of the methanol oxidation process at 0.7 V vs. Ag/AgCl, the Ni@PCFs-8 retained 82% of its initial current density, demonstrating its high stability. We believe that the strategy used in this study will open new prospects for the production of efficient and inexpensive monometallic electrocatalysts—thus contributing to the development of fuel cells in the future. However, the adsorption-pyrolysis method for the preparation of bi/tri metallic electrocatalysts needs further studies to design a highly selective hydrogel that adsorbs metals in specific proportions.

## 4. Materials and Method

### 4.1. Materials

Ammonium persulfate (APS), nickel acetate tetrahydrate, N,N’-Methylenebisacrylamide, Nafion solution (5% wt), isopropyl alcohol, acetone, diamond suspension and methanol were obtained from Sigma/Aldrich, Darmstadt, Germany and used without further purification. Chitosan was obtained from Poly-sciences, Inc., Warrington, PA, USA. Acrylic acid was purchased from QUALIKEMS Fine Chem Pvt. Ltd., Delhi, India. 

### 4.2. Synthesis of Hydrogel Nanocomposites

The hydrogel nanocomposite was prepared by an in situ grafting method, according to our previous study [44]. concisely, 0.5 g of chitosan was dissolved in acetic acid (30 mL, 1.0 percent *w*/*v*) in a three-necked conical flask with stirring at 25 °C. After complete chitosan dissolution, 0.075 g of ECNF powder was sonicated for 15 min and then heated at 60 °C with stirring and purged with nitrogen gas. After that, 0.1 g of APS was added to the mixture, and following this, 4 mL of acrylic acid and 0.075 g of N,N′-Methylenebisacrylamide were also added. The reaction was left for an hour at 60 °C under nitrogen gas, then cooled, washed with sodium hydroxide (0.1 M) and methanol, filtered, and dried at 50 °C.

### 4.3. Synthesis of Ni@PCFs Catalyst

In a typical preparation, 4 g of hydrogel nanocomposite was placed in a solution of nickel acetate tetrahydrate (0.033 M) and stirred at room temperature until the nickel ion adsorption on the hydrogel surface reached equilibrium, after 24 h. Then, the hydrogel nanocomposite/Ni^2+^ was filtered and dried at 60 °C for 24 h. The hydrogel nanocomposite/Ni^2+^ was pyrolyzed in a tubular furnace at 200 °C for 30 min, 300 °C for 1.0 h, and finally for 2.0 h at 600, 700 and 800 °C at a heating rate of 5 °C min^-1^ under nitrogen gas. The prepared samples were labeled with Ni@PCFs-6, Ni@PCFs-7, and Ni@PCFs-8 according to the pyrolysis temperature used.

### 4.4. Catalyst Evaluation

Initially, 5 mg of the catalyst was dispersed by ultrasonication in 400 μL of isopropyl alcohol for 0.5 h. Then, 20 μL of Nafion was added to the dispersed catalyst, with continuous ultrasonication for another 10 min. Then, immediately, 15 μL of the catalyst ink was deposited in three batches on the glassy carbon electrode surface (surface area; 0.07 cm^2^). The modified glassy carbon electrode (GCE) was then dried at room temperature for 24 h before being dried at 80 °C for 10 min under a vacuum. Electrochemical tests were performed by a potentiostat (VersaSTAT3, AMETEK-USA) using a three-electrode cell at 25 °C. The three-electrode cell consists of Ag/AgCl (3 M KCl) as the reference electrode, modified GCE as the working electrode and platinum wire as the counter electrode.

### 4.5. Characterization

Field-emission scanning electron microscopy (FE-SEM; JSM7100F, Tokyo, Japan) and transmission electron microscopy (TEM; JEM-2010, Tokyo, Japan) were used to examine the morphology and particle size of the obtained samples. X-ray diffraction (XRD; Bruker-D8 DISCOVER, Karlsruhe, Germany) and EDX analysis were used to investigate the chemical composition of the catalyst. Under a nitrogen atmosphere, thermogravimetric analysis (TGA; New-Castle, TA, USA) was used to investigate the thermal stability of the samples and to determine the best route for the calcination process.

## Figures and Tables

**Table 1:** XRD characteristics of the Ni@PCFs catalysts.

**Nickel**	**Peak Position** **(2 Theta)**	**FWHM**	**Crystallite Size (D)** **(nm)**	**Aver. of D (nm)**	**d-Space (nm)**
Ni@PCFs-6	44.50	0.89392	9.60	9.50	0.20343
	51.83	1.08946	8.11		0.17623
	76.42	0.93777	10.78		0.12453
Ni@PCFs-7	44.42	0.41449	20.70	15.21	0.20380
	51.77	0.49561	14.42		0.17646
	76.32	0.59526	10.49		0.12467
Ni@PCFs-8	44.32	0.34699	24.72	22.84	0.20421
	51.65	0.39374	22.42		0.17682
	76.11	0.47196	21.38		0.12497
**Carbon**	**Peak Position** **(2 Theta)**	**FWHM**	**% Graphitization**		**d-Space (nm)**
Ni@PCFs-6	24.78	6.37	0.00		0.35898
Ni@PCFs-7	25.95	1.76	10.23		0.34312
Ni@PCFs-8	26.12	1.36	36.86		0.34083

**Table 2:** Comparison of the current density of differnt nickel-based catalysts supported on carbon materials.

Catalyst	Conditions	Current Density (mA/cm^2^)	Ref.
Ni_2.5_Co_0.5_Sn_2_	CH_3_OH (2M)/KOH (1M), E = 0.6 V vs. Hg/HgO, ν = 0.1 V/s	65.5	[39]
Ni-NPs/RCQDs/GCE	CH_3_OH (2M)/KOH (1M), E = 0.56 V vs. Ag/AgCl, ν = 0.05 V/s	32	[20]
Ni NPs@r-GO	CH_3_OH (0.08M)/NaOH (0.11M), E = 0.536 V vs. Ag/AgCl ν = 0.1 V/s	20	[19]
Ni/C	CH_3_OH (0.6M)/KOH (0.5M), E = 0.735 V vs. Hg/HgO, ν = 0.01 V/s	22.13	[40]
NiCu@C	CH_3_OH (1M)/KOH (1M), E = 0.586 V vs. Hg/HgO, ν = 0.05 V/s	41.12	[41]
NiNPs@CFs	CH_3_OH (0.5M)/KOH (1M), E = 0.8 V vs. Hg/HgO, ν = 0.05 V/s	2.0	[21]
NiNPs/ITO	CH_3_OH (0.5M)/NaOH (0.1M), E = 0.71 V vs. Ag/AgCl, ν = 0.05 V/s	5.47	[42]
NiNP-GE	CH_3_OH (0.5M)/NaOH (0.5M), E = 0.8 V vs. SCE, ν = 0.1 V/s	7.0	[43]
**Ni@PCFs-6**	CH_3_OH (0.5M)/KOH (1M), E = 0.8 V vs. Ag/AgCl, ν = 0.05 V/s	34.58	**This work**
**Ni@PCFs-7**		74.16	
**Ni@PCFs-8**		158	

## Supplementary Materials

The following supporting information can be downloaded at: https://www.mdpi.com/article/10.3390/gels8090542/s1, Figure S1: SEM and TEM images of oxidized ECNFs; Figure S2. SEM images of Ni NPs@C-8; Figure S3. The EDX area of the hydrogel nanocomposite/Ni^2+^ and its corresponding data; Figure S4. The EDX area of the Ni@PCFs-8 catalyst and its corresponding data; Figure S5. Cyclic voltammograms at various concentration of methanol (**a**) Ni@PCFs-6, (**b**) Ni@PCFs-7, (**c**) Ni@PCFs-8 recorded at 0.05 V/s in 1.0 M KOH.
