# Supplementary material for: Hydrogel Nanocomposite-Derived Nickel Nanoparticles/Porous Carbon Frameworks as Non-Precious and Effective Electrocatalysts for Methanol Oxidation
Source: Gels. 2022 Aug 29;8(9):542. doi: 10.3390/gels8090542 (PMC9498779; doi:10.3390/gels8090542)
Supplement: Supplementary file 1 [file gels-08-00542-s001.zip › gels-1872446-supplementary.pdf]

Supporting Information

Hydrogel nanocomposite-derived nickel nanoparticles/porous carbon frameworks as non-precious and effective electrocatalysts for methanol oxidation

Hamud A. Altaleb [1], Abdulwahab Salah [2] and Badr M. Thamer [3,*]

[1] Department of Chemistry, Faculty of Science, Islamic University of Madinah, Madinah 42351, Saudi Arabia

[2] Faculty of Chemistry, Northeast Normal University, Changchun, China

[3] Chemistry Department, Science College, King Saud University, Riyadh, Saudi Arabia

* Correspondence: bthamer@ksu.edu.sa

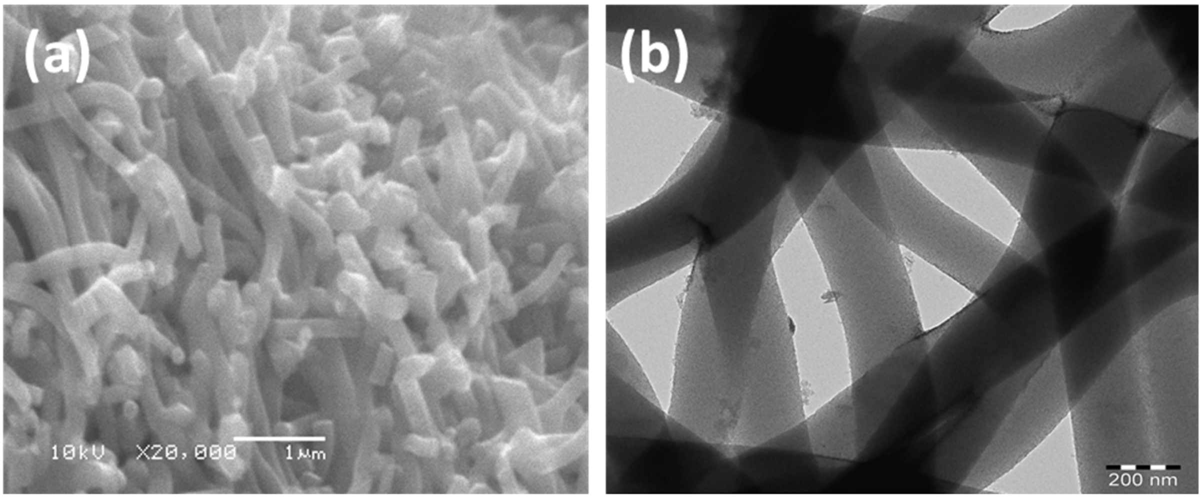

Figure S1. SEM and TEM images of oxidized ECNFs.

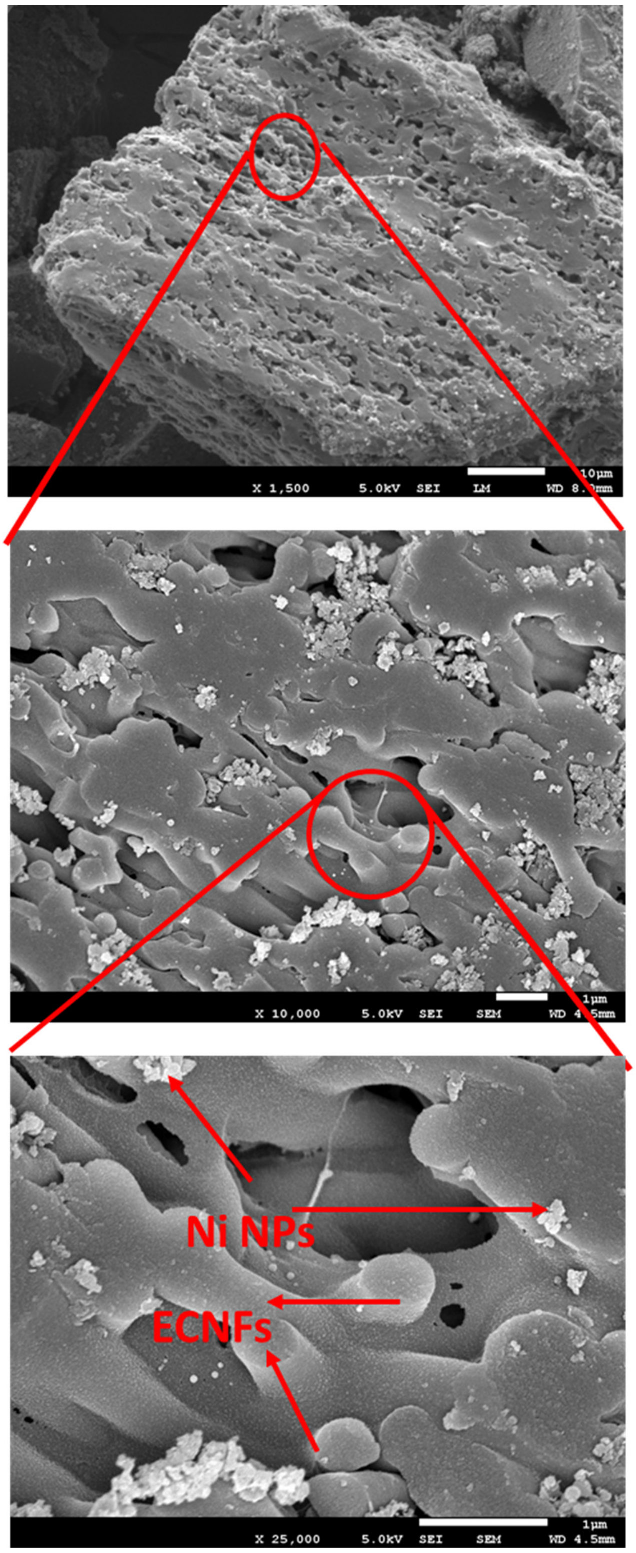

Figure S2. SEM images of Ni NPs@C-8.

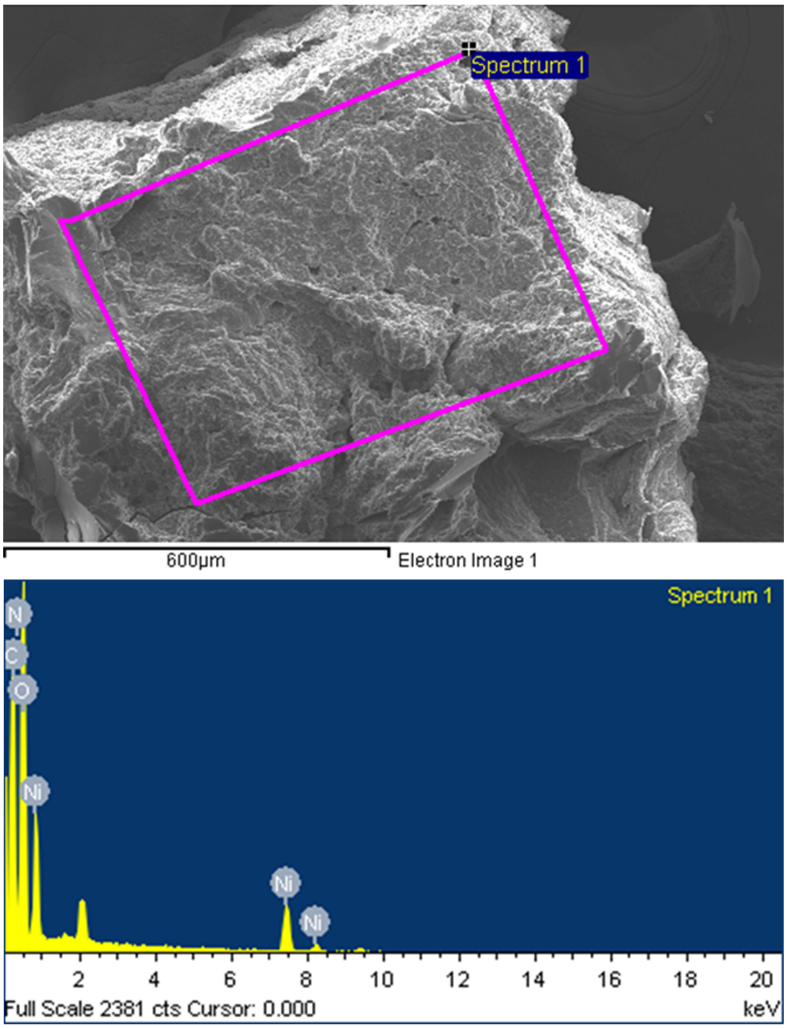

Figure S3. The EDX area of the hydrogel nanocomposite/Ni^{2+} and its corresponding data.

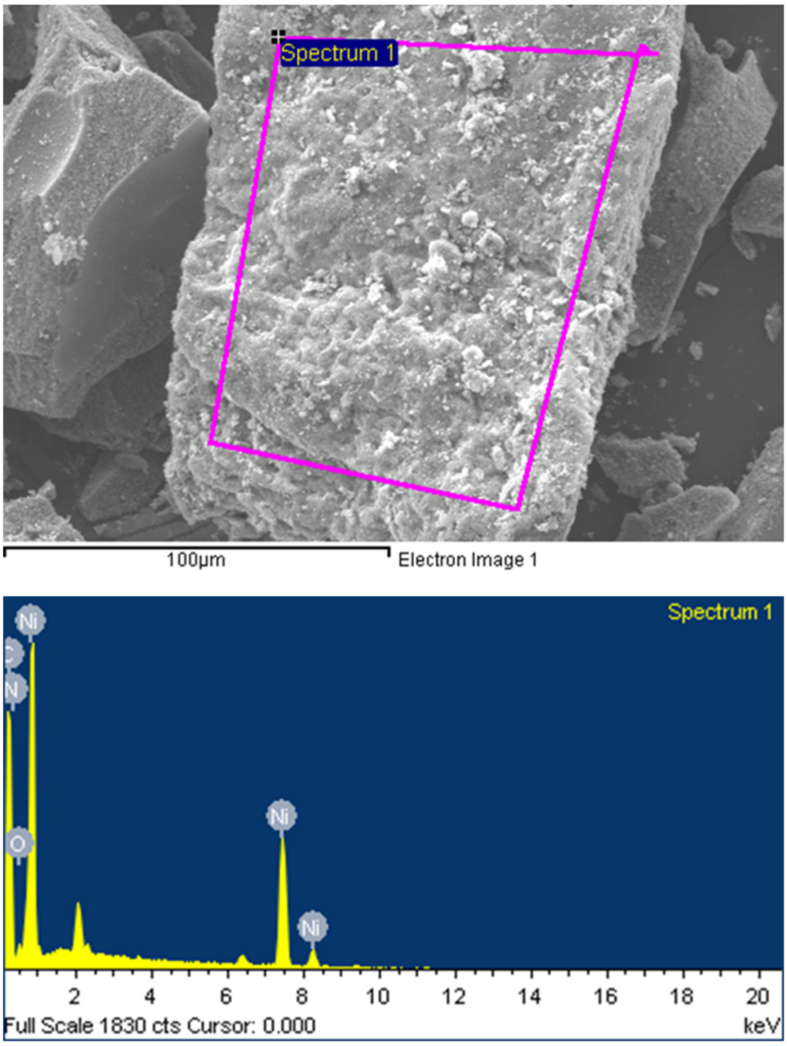

Figure S4. The EDX area of the Ni@PCFs-8 catalyst and its corresponding data.

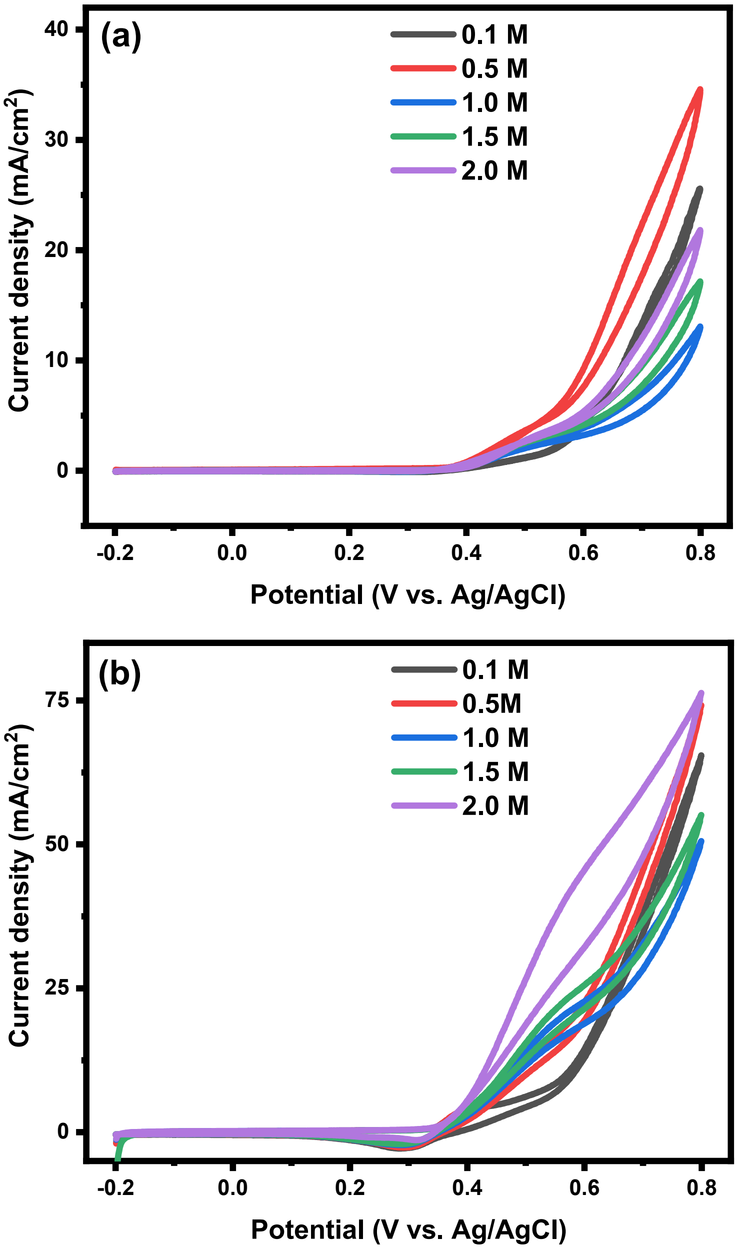
(a)
0.1 M
0.5 M
1.0 M
1.5 M
2.0 M
Current density (mA/cm^2)
40
30
20
10
0
-0.2
0.0
0.2
0.4
0.6
0.8
Potential (V vs. Ag/AgCl)
(b)
0.1 M
0.5M
1.0 M
1.5 M
2.0 M
Current density (mA/cm^2)
75
50
25
0
-0.2
0.0
0.2
0.4
0.6
0.8
Potential (V vs. Ag/AgCl)

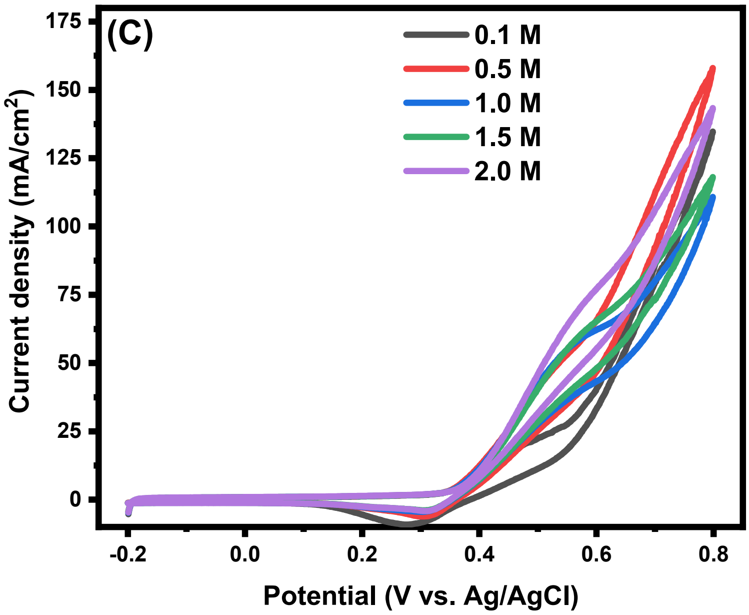

Figure S5. Cyclic voltammograms at different concentration of methanol (**a**) Ni@PCFs-6, (**b**) Ni@PCFs-7, (**c**) Ni@PCFs-8 recorded at 50 mV/s in 1.0 M KOH solution.
